# Revealing the Structural Coloration of Self‐Assembled Chitin Nanocrystal Films

**DOI:** 10.1002/adma.202203300

**Published:** 2022-06-28

**Authors:** Aurimas Narkevicius, Richard M. Parker, Jordi Ferrer‐Orri, Thomas G. Parton, Zihao Lu, Gea T. van de Kerkhof, Bruno Frka‐Petesic, Silvia Vignolini

**Affiliations:** ^1^ Yusuf Hamied Department of Chemistry University of Cambridge Lensfield Road Cambridge CB2 1EW UK

**Keywords:** chitin, liquid crystals, nanocrystals, self‐assembly, structural color

## Abstract

The structural coloration of arthropods often arises from helicoidal structures made primarily of chitin. Although it is possible to achieve analogous helicoidal architectures by exploiting the self‐assembly of chitin nanocrystals (ChNCs), to date no evidence of structural coloration has been reported from such structures. Previous studies are identified to have been constrained by both the experimental inability to access sub‐micrometer helicoidal pitches and the intrinsically low birefringence of crystalline chitin. To expand the range of accessible pitches, here, ChNCs are isolated from two phylogenetically distinct sources of α‐chitin, namely fungi and shrimp, while to increase the birefringence, an in situ alkaline treatment is performed, increasing the intensity of the reflected color by nearly two orders of magnitude. By combining this treatment with precise control over ChNC suspension formulation, structurally colored chitin‐based films are demonstrated with reflection tunable from blue to near infrared.

## Graphical abstract

Chitin nanocrystals can be sustainably extracted from mushrooms and shrimp shells. These elongated nanoparticles spontaneously organize in water to form a chiral nematic mesophase. Upon evaporation, this helicoidal architecture is preserved, resulting in structurally colored films. An alkaline post‐treatment to convert chitin into chitosan is found to significantly enhance the birefringence, resulting in vibrant red, green, and blue coloration.

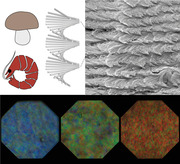

## 1 Introduction

To meet the growing demand for functional materials with tailored properties, the pursuit of optimal performance must be balanced against a consideration of the sustainability of the source materials.^[^ ^1^, ^2^ ^]^ One approach to address this challenge is to take inspiration from the natural world, where a limited selection of simple components can be assembled into various hierarchical structures to achieve diverse functionalities.^[^ ^3^ ^]^ This concept is well exemplified in arthropods, such as insects, spiders, and crustaceans, where numerous structural functions are achieved using chitin as the primary building block.^[^ ^4^ ^]^ The chains of this polysaccharide are assembled into fibrillar units, which are tightly bound together by a dense network of hydrogen bonds and van der Waals interactions.^[^ ^5^ ^]^ These microscale fibrils then form the building blocks for larger‐scale architectures. In particular, the arrangement of fibrils into a helicoidal configuration, where they are locally aligned with a twist perpendicular to the alignment axis, is a ubiquitous motif across the *Arthropoda* phylum.^[^ ^6^ ^]^ Notable examples are the dactyl clubs of the mantis shrimp,^[^ ^7^ ^]^ which incorporates a helicoidal architecture with large, micrometer‐scale periodicity (termed “pitch”) responsible for reducing crack propagation, or the cuticle of the scarab beetle,^[^ ^8^ ^]^ whose striking structural coloration arises from a helicoidal nanostructure with a pitch on the length scale of visible light (i.e., 250–450 nm).^[^ ^9^, ^10^ ^]^ However, despite being an abundant, biodegradable, and versatile biomaterial, the potential of chitin as a feedstock for bespoke optical materials has not yet been realized.^[^ ^11^, ^12^ ^]^

**Figure 1:**
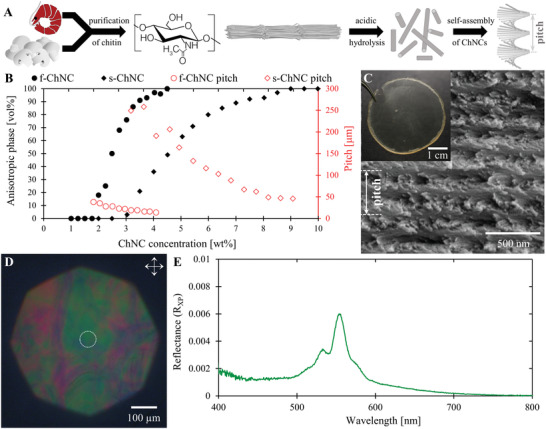
Structurally colored ChNC films produced via the self‐assembly of chitin nanocrystals. A) A schematic illustrating the extraction, formation, and self‐assembly of chitin nanocrystals. B) Proportion of anisotropic phase (black full symbols) and the pitch (red open symbols) for ChNC suspension derived from fungal chitin (f‐ChNC, circles) and shrimp chitin (s‐ChNC, diamonds). C) Cross‐sectional SEM image of a f‐ChNC film, showing a pitch (*p* = 360 ± 90 nm) compatible with structural color in the visible range. A photograph of the film is shown in the inset, which appears colorless by eye. D) Crossed polarized microscopy image in epi‐illumination of the f‐ChNC film revealing faint structural coloration. E) The corresponding microspectroscopy of the film (white dotted circle in (C)) confirming a weak, narrow peak in the reflection spectrum.

Helicoidal nanoarchitectures can be reproduced by exploiting the self‐assembly of chitin nanocrystals (ChNCs), which are elongated nanoparticles obtained by the acid hydrolysis of purified natural chitin.^[^ ^13^, ^14^, ^15^ ^]^ In acidic aqueous media, ChNCs spontaneously self‐organize into a chiral nematic (i.e., cholesteric) liquid crystalline phase (**Figure** **1**A).^[^ ^14^, ^15^, ^16^, ^17^ ^]^ The chiral ordering of this mesophase can be preserved as the suspension dries into the solid state.^[^ ^14^, ^15^, ^18^ ^]^ This self‐assembly process is tunable through both the properties of the ChNCs (e.g., surface charge, morphology, size) and the formulation of the medium (e.g., ionic strength, pH).^[^ ^15^, ^16^ ^]^ However, structural coloration in the visible range has not yet been reported for artificial chitin architectures^[^ ^11^ ^]^ because the smallest pitches reported so far are ≈650 nm.^[^ ^15^ ^]^

In this work, we demonstrate structural coloration in ChNC films by overcoming previous limitations in pitch and birefringence. To do so, we compare ChNCs extracted from two phylogenetically distinct sources of α‐chitin, namely from fungi (f‐ChNCs) and shrimp (s‐ChNCs). We observed that helicoidal f‐ChNC films can access the pitch range required for reflection across the visible spectrum. We then enhance the intrinsic birefringence within the helicoidal structure by employing an in situ alkaline treatment to convert chitin into chitosan, increasing the intensity of the reflected color by nearly two orders of magnitude. By combining this treatment with careful control over the formulation, we demonstrate photonic films that reflect red color when using s‐ChNCs, and blue, green, and red colors when using f‐ChNCs.

## 2 Results and Discussion

### 2.1 Self‐Assembly of ChNCs into Helicoidal Films

Fungal chitin was extracted from a fresh sample of the common mushroom *Agaricus bisporus*, while shrimp chitin was refined from commercial shrimp chitin powder (Fisher Scientific, practical grade). A series of similar purification steps were performed to obtain pure α‐chitin from each source (see the Experimental Section), with the purification process monitored by carbon‐13 solid state nuclear magnetic resonance (^13^C ss‐NMR) spectroscopy, powder X‐ray diffraction (p‐XRD), and attenuated total reflection Fourier transform infrared (ATR‐FTIR) spectroscopy (Figures S1–S3, Supporting Information). To produce ChNCs, the purified chitin was then subjected to hydrolysis with hydrochloric acid (3.0 m, 60 mL g_chitin_^−1^, 100 °C). Fungal chitin was hydrolyzed for 180 min as a longer duration resulted in significant discoloration, indicating the occurrence of undesirable side reactions, whereas shrimp chitin was carried hydrolyzed for 270 min (previously reported to be the optimal conditions^[^ ^15^ ^]^). The resultant f‐ChNC and s‐ChNC suspensions were diluted to 1.0 wt% and dialyzed against a hydrochloric acid solution ([HCl] = 0.6 × 10^−3^ m) until the conductivity of the dialysis bath was constant. The suspensions were then ultrasonicated, resulting in colloidally stable suspensions ([ChNC] = 1.0 wt%).

The self‐assembly of f‐ChNCs and s‐ChNCs was first investigated by preparing suspensions at a range of ChNC concentrations and observing their equilibrium phase behavior in glass capillaries. This revealed that while both suspensions could form a chiral nematic phase, their properties were significantly different (Figure 1B and Figure S4 (Supporting Information)). Although both suspensions were isotropic at a ChNC concentration of 1.0 wt%, the f‐ChNC suspension showed biphasic behavior (indicating liquid crystal ordering) from 1.75 wt%, compared to 3.0 wt% for the s‐ChNC suspension. A consequence of this earlier transition was that the f‐ChNC suspension formed a fully anisotropic chiral nematic phase at 4.5 wt%, while the s‐ChNC remained predominantly isotropic, with a fully anisotropic phase not reached till 9.0 wt%. The earlier onset of the chiral nematic phase in f‐ChNCs is consistent with their larger mean aspect ratio (as determined by transmission electron microscopy (TEM) and atomic force microscopy (AFM), Figure S5, Supporting Information), in accordance with Onsager's theory for lyotropic liquid crystals.^[^ ^19^ ^]^ Moreover, the chiral nematic phase of a f‐ChNC suspension had a significantly smaller pitch than a s‐ChNC suspension at comparable concentration, ionic strength, and pH, indicating a stronger chiral interaction between f‐ChNCs.^[^ ^15^, ^16^ ^]^ While the origin of a chiral mesophase in ChNCs suspensions is not well understood, recent studies on cellulose nanocrystals, a similar colloidal system, have demonstrated that the presence of crystallite bundles determines the chiral nematic pitch.^[^ ^20^, ^21^ ^]^ Qualitative observation of TEM images confirmed the presence of crystallite bundles (Figure S5, Supporting Information), but further quantitative analysis is required to confirm whether these bundles are also the origin of mesophase chirality in ChNC suspensions.

Photonic ChNC films were prepared by slow evaporation of aqueous suspensions of f‐ChNCs and s‐ChNCs. In both cases, cross‐sectional scanning electron microscopy (SEM) revealed the characteristic Bouligand arches expected for a helicoidal structure,^[^ ^22^ ^]^ with the pitch (*p*) measured as 360 ± 90 nm for f‐ChNCs (Figure 1C and Figure S6C (Supporting Information)) and 4.0 ± 1.9 µm for s‐ChNCs (Figure S6B, Supporting Information). The peak reflection wavelength of a helicoidal structure at normal incidence is given by Bragg's law (λ = *n*_avg_ *p*), where *n*_avg_ is the mean refractive index and is reported to be ≈1.55 for chitin.^[^ ^11^, ^23^, ^24^ ^]^ It is therefore expected that the pitch of the f‐ChNC film is in the range to reflect visible color, while the pitch of the s‐ChNC film is too large.^[^ ^14^, ^15^ ^]^ While both films appeared macroscopically transparent (e.g., Figure 1C inset), very weak coloration could be observed using polarized microscopy on f‐ChNC films (Figure 1D). When imaged between crossed linear polarizers in epi‐illumination, green and red regions could be observed. The former are consistent with the minimum pitch measured by SEM, while the latter are attributed to tilted domains that experience less axial compression upon drying and consequently have a larger final pitch, as previously been described for analogous structures.^[^ ^25^, ^26^ ^]^ An example SEM cross‐section showing tilted domains within a ChNC film is reported in Figure S14D (Supporting Information). A green region in the f‐ChNC film was characterized by microspectroscopy between crossed polarizers (Figure 1E, see the Experimental Section), which revealed a narrow reflection peak with very low reflectance (<1%) that is consistent with the lack of visible macroscopic coloration.

### 2.2 Tuning the Helicoidal Pitch of Photonic ChNC Films

**Figure 2:**
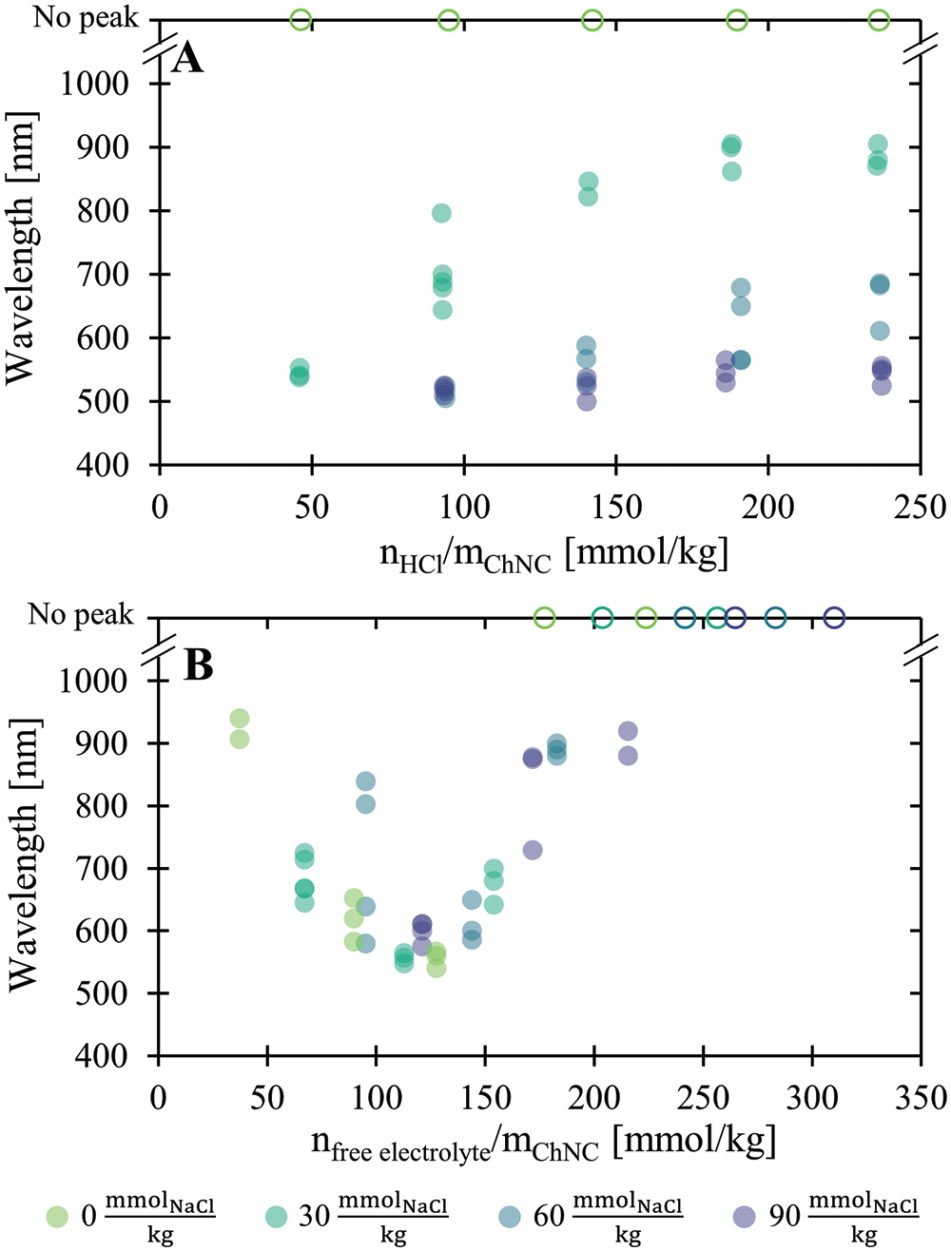
Tuning of the reflection peak wavelength of f‐ChNC films. A) Effect of the ratio of HCl to f‐ChNC, added after extensive dialysis against Milli‐Q water and the addition of increasing amounts of NaCl. B) Effect of additional monovalent ions (HCl or NaCl) to a fully protonated f‐ChNC suspension (achieved by prior addition of 246 mmol kg^−1^ of HCl). The full circles denote the wavelength of peaks reflection, while the open circles denote ChNC films where a reflection peak was not observed within the measurement window of the spectrometer.

The pitch of ChNC films can be tuned by varying the ionic strength and pH of the cast suspension (**Figure** **2**A,B).^[^ ^15^ ^]^ To explore this concept, the f‐ChNC suspension was extensively redialyzed against Milli‐Q water. During this process, the ionic strength of the suspension became extremely low and the pH tended to neutrality, leaving only a fraction of the surface amines protonated (p*K*_a_ = 6.3).^[^ ^14^ ^]^ Sodium chloride (NaCl) can then be added to increase the ionic strength without changing the level of protonation. Alternatively, addition of hydrochloric acid (HCl) first increases the degree of protonation of the surface‐bound amines. Once the surface is fully protonated, further addition of HCl simply contributes to the ionic strength, analogous to the effect of adding NaCl. The amount of HCl required to fully protonate an f‐ChNC suspension (expressed as moles of ions per ChNC dry mass) was determined to be 246 mmol kg^−1^ by conductometric titration (Figure S7, Supporting Information), consistent with the observed significant decrease in viscosity of the f‐ChNC suspension above 250 mmol kg^−1^ of added HCl. As the surface‐bound amines become protonated, the overall charge of the nanoparticles increases, which was found to redshift the reflected color of the resultant f‐ChNC films (Figure 2A). Once all the surface amines are protonated (i.e., [HCl] > 246 mmol kg^−1^), addition of further monovalent electrolyte (HCl or NaCl) results in a subsequent blueshift in the color of the f‐ChNC film (Figure 2B), followed by a reversal toward red wavelengths beyond 150 mmol kg^−1^ of additional electrolyte. By controlling the initial formulation of the f‐ChNC suspension in terms of the amounts of HCl and/or NaCl added, f‐ChNC films that reflect wavelengths ranging from 550 nm (green) to 1000 nm (infrared) could be obtained. Similarly, by controlling the initial formulation of the s‐ChNC suspension, the pitch could be reduced to the smallest value of 648 ± 77 nm, still too large for visible structural coloration (Figures S8A–C and S9E,F (Supporting Information)). Microspectroscopy in the infrared region of the s‐ChNC films confirmed reflection peaks at various wavelengths above 850 nm arising from domains with different pitches and orientations (Figure S8G, Supporting Information). While the values of pitch in the s‐ChNC and f‐ChNC films are clearly different, the intensities of the reflection peaks are of the same order of magnitude, suggesting that their local optical properties (e.g., birefringence, alignment) are broadly consistent.

### 2.3 Elucidating the Optical Properties of Photonic ChNC Films

**Figure 3:**
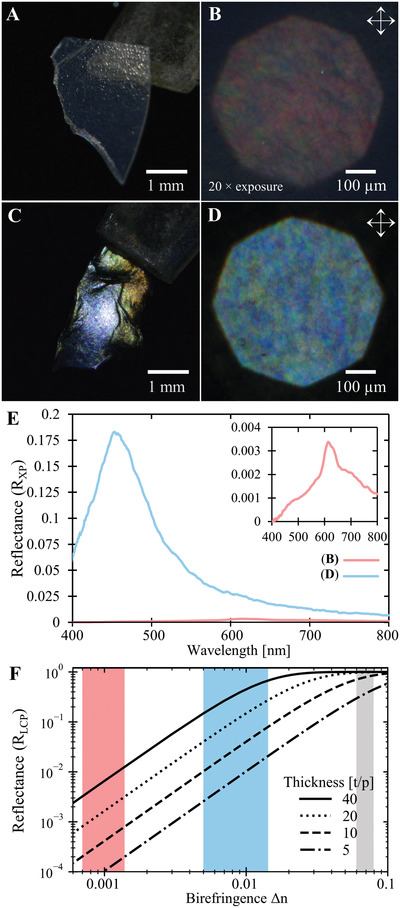
Enhancing structural coloration by alkaline post‐treatment of ChNC films. A) Photograph of an apparently colorless flake from an f‐ChNC film. B) Optical microscopy of the flake in (A) using crossed linear polarizers. Increasing the exposure 20 × reveals the presence of weak red/green structural coloration. C) Photograph of a f‐ChNC film flake after alkaline treatment, exhibiting strong blue/green coloration. D) Optical microscopy image of the flake in (C), confirming higher reflectance and a blueshift of the reflected color. E) Reflection spectra under crossed polarizers corresponding to (B) and (D) showing the weak red–green reflectance for the original f‐ChNC film (red line, enlarged in inset spectrum) and the more intense blueshifted reflectance after alkaline post‐treatment (blue line). F) The maximum reflectance for helicoidal structures with varied thicknesses and birefringence, calculated using de Vries theory. Estimated birefringence ranges are shown as shaded areas for the untreated f‐ChNC film (red) and the alkaline treated film (blue). For comparison, the estimated birefringence range for photonic cellulose nanocrystal (CNC) films is also indicated (gray).

The weak reflectance of the ChNC films can be explained by considering the birefringence of chitin and the dimensions of individual domains. By modeling a left‐handed helicoidal structure as a stack of birefringent layers with a fixed rotation angle between each layer, it can be shown that for structures with small birefringence relative to the average refractive index (Δ*n* ≪ *n*_avg_), the peak reflectance of left‐circularly polarized (LCP) light at normal incidence is given by

(1) $$R_{\text{LCP}}=\tanh^{2}\left(\pi \Delta nt/n_{\text{avg}}p\right)$$

where *t* is the domain thickness and *p* is the pitch (see the Supporting Information for extended derivation based on the work of de Vries).^[^ ^23^ ^]^ This equation shows the importance of birefringence for achieving high LCP reflectance, as illustrated by **Figure** **3**F.

Experimentally, the LCP reflectance of the chiral nematic structure is hard to distinguish from the broadband specular reflection at the film–air interface due to its low intensity. We therefore measured the reflectance of the film in crossed polarizer configuration, *R*_XP_, (Figure 1E) to isolate the contribution from the helicoidal structure. To compare this theoretical prediction to experimental values, the ChNC domain thickness was first estimated by cross‐sectional SEM, which indicated *t*/*p* values up to 40 (Figure S14, Supporting Information). The reflectance of f‐ChNC films was then measured by microspectroscopy and found to have a maximum value of *R*_XP_ = 0.0036 (Figure S15A, Supporting Information). Using Equation (1) and assuming the brightest reflection comes from the largest domains, these values suggest an extremely small effective birefringence of ChNCs of Δ*n* ≈ 0.0007–0.0015 (indicated by the red shaded area in Figure 3F), consistent with previous estimates of the birefringence of α‐chitin in natural and synthetic structures.^[^ ^8^, ^27^ ^]^ For context, cellulose nanocrystals (CNCs), which are known to produce intense structural coloration from analogous helicoidal films, have a much higher effective and intrinsic birefringence (Δ*n*_CNC eff_ = 0.062 and Δ*n*_CNC_ = 0.081, respectively).^[^ ^28^, ^29^, ^30^ ^]^ The difference between the two values is related to the local alignment of individual nanocrystals within the helicoidal structure (see Discussion S2 in the Supporting Information).^[^ ^31^ ^]^ As indicated by the gray shaded area in Figure 3F, the much greater birefringence of photonic CNC films leads to near‐saturated reflection for *t*/*p* > 10. To attain a comparable reflectance (e.g., *R*_LCP_ > 0.9) from a ChNC film with a pitch of 400 nm and assuming the upper limit of our birefringence range, the thickness of a single uniform domain would still need to exceed 240 µm (i.e., *t*/*p* > 600), which is experimentally near‐impossible to achieve. In addition, while the imaginary refractive index of chitin is small (≈3.0 × 10^−4^)^[^ ^32^ ^]^ and thus absorption can be neglected for the films studied in this work (i.e., 20–40 µm thick), it is expected that absorption would become significant for films in this thickness range (i.e., >240 µm). These findings highlight the importance of increasing the birefringence of the nanorods, rather than aiming for thicker monodomain films, to significantly increase the film reflectance.

### 2.4 In Situ Conversion of Chitin to Chitosan

It is well established that exposure of cleaned crab and shrimp shells to strong base at elevated temperatures produces structural color.^[^ ^11^, ^33^, ^34^ ^]^ This artificial treatment not only removes protein from the chitinaceous cuticles, but also can convert chitin into its derivative, chitosan (Figure S10, Supporting Information). Given the broad and strong reflectance peaks achieved by this method, we postulate that the enhancement of the visual appearance must arise from an increase in the intrinsic birefringence. With this in mind, to enhance the birefringence of our ChNC films, we applied an alkali treatment (50 wt% NaOH, 90 °C, 8 h), to successfully convert chitin into chitosan, as verified by ATR‐FTIR spectroscopy (Figure S11, Supporting Information).^[^ ^35^ ^]^ When imaged by polarized optical microscopy, fragments of an f‐ChNC film with and without alkaline treatment reveal a dramatic 50‐fold enhancement in the reflected intensity and a substantial blueshift in reflected color, as exemplified in Figure 3A–E and Figure S15 (Supporting Information). The decrease in pitch correlates with the 10–20% reduction in film thickness (Figure S9, Supporting Information) and is attributed to the removal and solubilization of the acetyl groups, which represent ≈20% of the molecular weight of chitin. Therefore, assuming that the helicoidal domain distribution (i.e., *t*/*p*) has not changed, as the material retains the shape throughout the treatment, the only explanation for this significant increase in coloration is an increase in intrinsic birefringence upon conversion from chitin to chitosan. Considering the 50‐fold increase of reflectance (*R*_XP_ ≈ 0.175) and assuming no substantial change of the average refractive index of the films, we estimate from Equation (1) the effective birefringence of the chitosan‐converted films as Δ*n*_deAc_ ≈ 0.005–0.015, i.e., an order of magnitude increase. From this estimation, the minimum domain thickness to approach saturating reflection (*R*_LCP_ >  0.9) is only 20 µm (i.e., *t*/*p* ≈ 60), which is comparable to the largest domains observed in our SEM analysis. Finally, the prominence of the LCP peak reflectance reported in Figure S12E (Supporting Information) (*R*_LCP_ ≈ 0.3) is consistent with this estimation, as a domain thickness of *t*/*p* ≈ 40 predicts a birefringence of Δ*n*_deAc_ ≈ 0.008 (see blue shaded area in Figure 3F).

**Figure 4:**
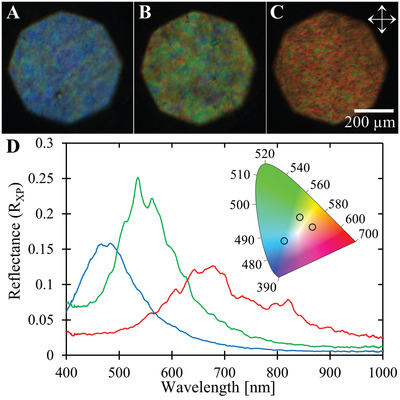
Tuning the structural coloration of post‐treated f‐ChNC films. A–C) Alkaline treatment on f‐ChNC films cast with 235 mmol_HCl_ kg^−1^ and 90, 60, or 30 mmol_NaCl_ kg^−1^, produced structurally colored films displaying (A) blue, (B) green, and (C) red color, respectively. D) Reflection spectra between crossed polarizers for the films in (A–C). Inset: CIE 1931 chromaticity diagram. The open circles denote the positions of the blue, green, and red films, with respective *x*–*y* coordinates of (0.21, 0.27), (0.33, 0.44), and (0.42, 0.37).

The combination of f‐ChNC self‐assembly and alkaline post‐treatment was then exploited to produce structurally colored films across the visible spectrum (**Figure** **4**). The alkaline treatment has the dual effect of reducing the helicoidal pitch and increasing birefringence, so a weakly colored green film (Figure 1D) becomes a vivid blue film after treatment (Figure 4A). Green and red f‐ChNC photonic films can analogously be produced by tuning the formulation of the initial suspension (Figure 4B,C, see the Experimental Section). The stronger reflectance of the post‐treatment films was confirmed by reflection microspectroscopy (Figure 4D), and their color purity visualized using an International Commision on Illumination (CIE) 1931 chromaticity diagram (inset in Figure 4D). For the s‐ChNC films, the significant reduction in pitch upon conversion from chitin to chitosan is crucial for visible coloration, with the initially weak infrared film displaying a strong red coloration after treatment (Figures S8 and S9E–H, Supporting Information). Furthermore, while the colored films are stable under ambient conditions, they are both biodegradable and readily dissolved in weakly acidic conditions, enabling the recycling of this material for other uses, e.g., fertilizer.^[^ ^36^ ^]^ Ultimately, the ability to tune the color from blue to infrared exemplifies the potential of ChNCs for sustainable photonic materials.

### 2.5 Comparison with Natural Chitinaceous Helicoidal Structures

Beyond the self‐assembly of photonic films from ChNCs, these studies offer some insight into their naturally occurring analogs. Numerous species of beetle employ chitinaceous helicoidal structures in their cuticles to produce brilliant structural color. It is perhaps surprising, therefore, that the untreated ChNC films in this work exhibit only a faint optical response, indicating that the intrinsic birefringence of α‐chitin alone is insufficient to explain the vibrant coloration observed from natural structures. Insect cuticles contain only ≈30% of chitin by composition, with the remainder being mostly made up by proteins, which suggests that the overall birefringence must be enhanced by the distribution of these other chemical components.^[^ ^6^ ^]^ Attempts here to replicate this effect via the addition of poly(ethylene glycol) into the ChNC film (Figure S16, Supporting Information) did not significantly increase the intensity of the reflected color, which could be attributed to the small refractive index contrast (Δ*n* < 0.1) and/or insufficient amount of additive (limited to less than 20 wt% to remain in the visible regime). Alternatively, the intense reflection observed in some beetle species (e.g., *Plusiotis resplendens* or *Plusiotis optima*) relies on the incorporation of highly birefringent uric acid within the helicoidal structure,^[^ ^8^ ^]^ suggesting that the chitin nanostructure mainly acts as a template in these species. By contrast, the conversion of chitin into chitosan employed in this work is unlikely to explain the optical response of natural helicoidal structures, as chitosan is not typically found in the arthropod cuticle in significant quantities.

## 3 Conclusions

Structurally colored chitin nanocrystal films with tunable color across the visible spectrum were successfully fabricated using fungal ChNCs. The distinctive mesophase behavior of f‐ChNCs gave access to much smaller helicoidal pitches in the solid state when compared to shrimp ChNCs. The very low reflectance of the pristine ChNC films (<1%) was overcome by an alkaline post‐treatment, which converts chitin to chitosan, causing both a blueshift in the reflected wavelength (10–20%) and an increase of the reflectance by nearly two orders of magnitude. The former is attributed to the removal of acetyl moieties throughout the volume of the ChNC nanoparticles, while the latter can be attributed an order‐of‐magnitude increase in the local birefringence upon conversion of chitin into chitosan. Our findings on the optical properties of the fabricated films offer an insight into how nature combines nanoarchitecturing and biomaterials to produce structural coloration; it suggests that natural systems often use composites to expand upon hierarchical motifs to enhance the overall optical performance of the material. The methods presented in this work provide a way to harness chitin, a renewable and abundant bioresource, for sustainable and biodegradable photonic materials.

## 4 Experimental Section

All reagents were of analytical grade and purchased from Fisher Chemicals (FSH International, USA), unless stated otherwise. Milli‐Q water was used throughout this work (Synergy UV water purification system, Merck, Germany). Shrimp chitin of practical grade, already partly purified from shrimp shells, was obtained from Fischer Scientific.

### Isolation of Chitin

To isolate fungal chitin from mushroom biomass, a modified extraction procedure was used.^[^ ^37^ ^]^ Common white mushrooms (*A. bisporus*, acquired from a local supplier) were washed under warm water, before boiling in water for 1 h and finally collecting with a cheese cloth. The cleaned mushrooms were then blended in water using a household stick blender, yielding a purée, which was further boiled in water for an hour. The solids were then collected and pressed again through a cheese cloth to yield a brownish‐gray pulp, which was then chemically treated by a two‐step alkaline‐bleach process. i) 100 g of the pulp was treated with aqueous NaOH (3 m, 200 mL) and NaBH_4_ (0.5 wt%, Acros Organics) at 80 °C for 3 h. The reaction mixture was subsequently centrifuged (25 000*g*, 30 min, 4 °C, Sorvall Lynx 6000) and the obtained pellet dispersed in Milli‐Q water. This washing procedure was repeated 3 times. ii) 100 g of this pellet was treated with 300 mL of hydrogen peroxide solution (2.5 wt%, pH ≈ 11, set with NaOH) at 80 °C for 2 h. The solids were collected and purified by centrifugation, as above. The steps (i) and (ii) were repeated once more, followed by collecting the purified chitin by vacuum filtration. The chitin was washed with Milli‐Q water to afford a wet white cake. Purification was concluded with dialysis against Milli‐Q water for 5 days after which the chitin was stored at 4 °C.

### Preparation of ChNC Suspensions

f‐ChNCs were obtained using a modified version of a previously reported procedure.^[^ ^15^ ^]^ The purified fungal chitin (10 g) was refluxed in aqueous HCl solution (3 m HCl, 60 mL g_dry‐chitin_^−1^, 100 °C) for 180 min. The reaction was then quenched by threefold dilution with ice‐cold water. The solids were collected by centrifugation (25 000*g*, 30 min, 4 °C), and dispersed in Milli‐Q. This washing step was repeated followed by dialysis for 5 days against Milli‐Q water. The resulting dilute fungal ChNC suspension (1.0 wt%) was then dialyzed against aqueous HCl solution (0.6 × 10^−3^ m) until the conductivity in the dialysis bath stopped changing overnight. The suspension was then tip sonicated (6.75 s mL^−1^, at 1 wt%) using 30% amplitude (Fischer Sonic Dismembrator, 500 W) and vacuum‐filtered (8.0 and 0.8 µm nitrocellulose filter paper, Millipore, Germany). The f‐ChNC suspension was then concentrated to 8.81 ± 0.06 wt%, using a rotary evaporator operating at 50 °C. A drop of chloroform was added to prevent bacterial growth and the suspension was stored at 4 °C prior to use.

The s‐ChNC suspension was prepared according to a previously reported method.^[^ ^15^ ^]^ Briefly, the shrimp chitin was hydrolyzed in 3.0 m HCl for 270 min at reflux and the resulting suspension was collected by centrifugation (25 000*g*, 30 min, 4 °C). The pellet was redispersed in Milli‐Q and centrifugation applied again and dialyzed against Milli‐Q. The ionic strength and pH were set by dialysis of 1.0 wt% ChNC suspension against 0.6 × 10^−3^ m aqueous HCl solution. The s‐ChNC suspension was then concentrated to 12.87 ± 0.05 wt%, using a rotary evaporator operating at 50 °C. A drop of chloroform was added to prevent bacterial growth and the suspension was stored at 4 °C prior to use.

Note that for the study of the effects of added NaCl and HCl on the color of the films (Figure 2), the suspensions were instead extensively dialyzed against Milli‐Q water, followed by dilution with appropriate amounts of NaCl and HCl solution.

### Evaluation of Liquid Crystalline Behavior of ChNC Suspensions

The liquid crystalline behavior was investigated by preparing a dilution series of f‐ChNC suspension (1.00–5.00 wt%) and loading into a set of round glass capillaries (1.3 mm inner diameter). The capillaries were then sealed using wax and left standing vertically and undisturbed for two weeks, after which they were photographed (Nikon D3200, AF‐S DX NIKKOR 18–55 mm f/3.5–5.6G VR lens) by placing the capillaries between crossed linear polarizers with rear illumination. The volume percent of the anisotropic phase was defined as the ratio of the height of the anisotropic phase (which appears bright in photographs), over the total height of the suspension (Figure S4A, Supporting Information). These capillaries were then examined by polarized optical microscopy in transmission mode with the sample placed between crossed linear polarizers (Nikon SLWD 20×/0.30, Zeiss Axio inverted microscope). All the images were analyzed using ImageJ software. The pitch of the chiral nematic phase, as measured by optical microscopy, was defined as twice the periodicity of the fingerprint pattern (Figure S4B, Supporting Information). The mean value was calculated from several images per capillary.

### Casting of Photonic ChNC Films

To obtain ChNC films, suspensions at 2.0 wt% (3.0 g of wet mass for f‐ChNC and 4.0 g for s‐ChNC) were dried in polystyrene (PS) Petri dishes (35 mm diameter, nontreated PS, ref. 430588, Corning VWR, USA) at room temperature over several days in a ventilated cupboard, unless otherwise stated. Generally films did not stick to the Petri dish and could be peeled off.

### Conversion of Chitin to Chitosan

Flakes of ChNC films were converted to chitosan by immersing ChNC in an aqueous NaOH solution (50 wt%, 90 °C, 8 h). Treated ChNC film flakes were then extensively washed with Milli‐Q water until at neutral pH and left to dry under ambient conditions in polystyrene Petri dishes. The flakes generally stuck to the Petri dish and were difficult to remove. Using nonstick hydrophobic surfaces was not found to be beneficial, as the drying films curled up. As a control, applying an equivalent thermal treatment in water did not induce any notable changes to ChNC films.

### Optical Characterization of Photonic Films

The optical response of the ChNC films was characterized using a custom optical microscope (Axio Scope A.1, Zeiss) in bright‐field reflection (epi‐illumination) mode. Crossed polarization (XP) measurements were collected between two linear polarizers (WP25M‐UB, Thorlabs). Left and right circular polarization measurements were collected by filtering reflected light through a quarter‐wave plate (B‐Halle) followed by a linear polarizer (WP25M‐UB, Thorlabs). Images and spectra were collected from flakes originating from the central regions of films to avoid coffee‐ring edge effects. All microscopy images were obtained using an IDS camera (UI‐3580LE, IDS) with a 10× objective (Zeiss EC Epiplan‐Apochromat 10×/0.3 HD DIC 422642‐9960). The color balance was set against a standard white diffuser (USRS‐99‐010, Labshare). Microspectroscopy was typically performed by collecting reflected light using an optical fiber (core diameter 200 µm, FC‐UVIR200‐2, Avantes) in confocal configuration, which was coupled to a spectrometer (AvaSpec‐HS2048, Avantes). For Figure 2, a wider optical fiber was used (core diameter 1000 µm, FC‐UV1000‐2‐SR, Avantes) to obtain the optical response over a larger area. Spectra were normalized to a silver mirror (PF10‐03‐P01, Thorlabs). All the presented optical spectra were smoothed via a moving average of the nearest 9 neighbors, corresponding to a wavelength window of 5 nm. CIE 1931 chromaticity diagram was calculated from XP spectra using the Color package (v. 0.4.1) in Python.^[^ ^38^ ^]^ Photographs were obtained using a Nikon camera (D3200, AF‐S DX NIKKOR 18–55 mm f/3.5–5.6G VR lens).

### Structural Characterization of Helicoidal Films

Samples for scanning electron microscopy were prepared by breaking films into flakes by laterally pulling them apart to favor crack propagation. The flakes were mounted on an aluminum stub using carbon tape and sputter coated with 10 nm of Au/Pd (Quorum Q150T ES). Imaging was performed using MIRA 3 Scanning electron microscope (Tescan) scanning electron microscope, operating at high vacuum mode at 3–5 kV with a 3–5 mm working distance using in‐beam SEM detector. The pitch was measured directly from the cross‐sectional images and is reported as the mean value and the standard deviation.

## Conflict of Interest

The authors declare no conflict of interest.

## Supporting information

Supporting Information

## Data Availability

The data that support the findings of this study are openly available in the University of Cambridge data repository at https://doi.org/10.17863/CAM.85268.
