# Supplementary material for: Revealing the Structural Coloration of Self‐Assembled Chitin Nanocrystal Films
Source: Adv Mater. 2022 Jun 28;34(31):2203300. doi: 10.1002/adma.202203300 (PMC11475362; doi:10.1002/adma.202203300)
Supplement: Supplementary file 1 — Supporting Information [file ADMA-34-2203300-s001.pdf]

ADVANCED MATERIALS

Supporting Information

for *Adv. Mater.,* DOI: 10.1002/adma.202203300

# Revealing the Structural Coloration of Self-Assembled Chitin Nanocrystal Films

*Aurimas Narkevicius, Richard M. Parker, Jordi Ferrer-Orri, Thomas G. Parton, Zihao Lu, Gea T. van de Kerkhof, Bruno Frka-Petesic, and Silvia Vignolini**

# Supporting Information

**Revealing the Structural Coloration of Self-Assembled Chitin Nanocrystal Films**

*Aurimas Narkevicius, Richard M. Parker, Jordi Ferrer-Orri, Thomas G. Parton, Zihao Lu, Gea T. van de Kerkhof, Bruno Frka-Petesic, and Silvia Vignolini**

Dr. Aurimas Narkevicius, Dr. Richard M. Parker, Jordi Ferrer-Orri, Thomas G. Parton, Zihao Lu, Dr. Gea T. van de Kerkhof, Dr. Bruno Frka-Petesic, Prof. Silvia Vignolini

Yusuf Hamied Department of Chemistry, University of Cambridge, Lensfield Road, Cambridge, CB2 1EW, United Kingdom

E-mail: sv319@cam.ac.uk

## S1. Extended Experimental Methods

### Transmission Electron Microscopy (TEM)

The samples were prepared by depositing a drop of dilute ChNC suspension (0.001 wt%) on a glow-discharged carbon-coated copper grid. After 90 seconds, the excess liquid was blotted using filter paper, followed by staining with uranyl acetate (aqueous, 2.00 wt%) for 90 seconds. The grid was dried in air and then observed using either (i) a Tecnai G2 transmission electron microscope (FEI/ThermoFisher Scientific) operating at 200 kV and using a 20 mm objective aperture to improve contrast, with an ORCA HR CCD camera (AMT Corp.) for micrographs or (ii) Talos F200X G2 scanning transmission electron microscope (FEI/ThermoFicher Scientific) operating at 200 kV and a CCD camera.

### Atomic Force Microscopy (AFM)

AFM images were obtained by incubating a fully wetting droplet (dilute ChNC suspension of *ca.* 0.001 wt%) on freshly cleaved mica for 2 minutes, then rinsing with Milli-Q water and drying first with a flow of $N_2$ and then in an oven at 50 ºC for 1 hour. The measurements were obtained using a scanning probe microscope (Agilent 5500 SPM) in tapping mode with a commercial AFM probe (OTESPA-R3). The 4 x 4 µm size areas were scanned at a resolution of 1024 points per line, with a rate of 0.6 Hz. The data was processed using Gwyddion 2.8 software.

### Solid-state nuclear magnetic resonance spectroscopy (ssNMR)

Cross-polarization magic angle spinning $^{13}$C-ssNMR spectroscopy was performed at room temperature using an Ultrashield 400 spectrometer (Bruker) with a rotor spinning at 14 kHz. Glycine was used as a reference. A relaxation delay of 2 s and a contact time of 1 ms were used for the acquisition of spectra. The data was smoothed using the Savitzky-Golay method of third-degree polynomial with a window size of 51. Samples were freeze-dried before analysis.

### Powder X-ray Diffraction (pXRD)

The diffractograms were recorded using freeze dried material at angles (2θ) from 5º to 50º at 40 kV, 30 mA using an Empyrean powder diffractometer (Malvern Panalytical). The data was smoothed using the Savitzky-Golay method of third-degree polynomial with a window size of 51.

### FTIR spectroscopy

The spectra were collected in the 600-4000 cm$^{-1}$ range with 64 repeats and a resolution of 4 cm$^{-1}$ using a Spectrum 100 ATR spectrometer (PerkinElmer). The measurements were collected on freeze-dried samples.

### Conductometric Titration

For the data showed in Figure S8A, conductometric titration was performed manually by dropwise addition of HCl (10 mM, Titripur) into a diluted ChNC suspension (ca. 0.1 wt%). The amount of added acid each time was measured from the mass loss in the syringe containing the acid. The conductivity was determined following each addition (InLab 752-6mm electrode, Mettler Toledo). For the data showed in Figure S7B, conductometric titration of f-ChNC suspension was performed after it was fully protonated with an excess of added HCl. This titration was performed using NaOH (10 mM, Titripur) using an automated system (dosing unit 807, conductivity module 856, Metrohm).

### Casting of ChNC-PEG films

ChNC-PEG films were prepared by casting a formulation containing f-ChNC suspension (6.0 g at 1 wt%), HCl (328 mmol/g) and NaCl (30 mmol/g) and an appropriate amount of PEG (35 kDa) so that the final films would contain 0 – 20 wt% PEG.

**S2. Discussion of reflection from a thin helicoidal structure**

An ideal helicoidal structure of infinite thickness has a theoretical limit of 50% reflectance of unpolarized light due to the chirality of the helicoidal nanostructure (i.e., 100% LCP reflectance and 0% RCP reflectance for an ideal photonic ChNC film). As such, they can be outperformed by achiral photonic crystals (e.g., self-assembled colloidal opals or multilayers produced by top-down fabrication), which can reflect up to 100% of unpolarized light. Nevertheless, selective reflection of up to 50% of incident unpolarized light is sufficient to produce vibrant coloration.

In practice, the reflectance from helicoidal ChNC films depends on their (finite) thickness, pitch and effective birefringence. As such, the peak LCP reflectance from a thin helicoidal structure can be calculated using Equation 1 in the article. This equation is corollary of the work of de Vries,[1] but is not explicitly stated in that work, so a derivation is provided below using the same notation.

The optical response of a helicoidal structure can be predicted by modelling the structure as a stack of birefringent layers with a constant twist angle between each layer. In a given layer, the permittivity along the principal axes are $\varepsilon_1$ and $\varepsilon_2$, from which we define the average permittivity

$$\varepsilon = \frac{1}{2}(\varepsilon_1 + \varepsilon_2)$$

and a relative birefringence $\alpha$, given by

$$\alpha = \frac{\varepsilon_2 - \varepsilon_1}{2\varepsilon}.$$

By solving the electromagnetic boundary conditions between layers, it can be shown that the modes of a helicoidal multilayer with pitch $p$ are elliptically polarized, with an effective "refractive index" parameter $m$, given by Equation 12 in the work of de Vries[1]:

$$m^4 - 2m^2(\varepsilon + \lambda^2/p^2) + (\varepsilon_1 - \lambda^2/p^2)(\varepsilon_2 - \lambda^2/p^2) = 0$$

This equation can be made dimensionless by defining $m' = m/\sqrt{\epsilon}$ and relative wavelength $\lambda' = \lambda/(p\sqrt{\epsilon})$:

$$m'^4 - 2m'^2(1 + \lambda'^2) + (1 - \lambda'^2 - \alpha)(1 - \lambda'^2 + \alpha) = 0$$

The ellipticity of the polarization of the corresponding electromagnetic mode is given by

$$f = \frac{1 - \lambda'^2 - \alpha - m'^2}{2m'\lambda'}.$$

It is also convenient to define a factor $q = m' + \lambda' f$. The equation for $m'$ above can be considered a quadratic equation in $m'^2$ with two possible solutions (the equation is quartic in $m'$, but the difference between $\pm|m'|$ for each solution is only a change in the propagation direction). Explicitly, $m'^2$ is given by

$$m'^2 = (1 + \lambda'^2) \pm \sqrt{(1 + \lambda'^2)^2 - (1 - \lambda'^2 - \alpha)(1 - \lambda'^2 + \alpha)}$$

When considering the reflection from the helicoidal structure, the smaller solution ($m_1'^2$) is more relevant, as it takes negative values in the bandgap region leading to strong reflection, whereas the larger solution $m_2'^2$ is always positive.

The LCP reflectance from a left-handed helicoidal layer of thickness $t$ and pitch $p$ at normal incidence is given by the following equation:

$$R_{LCP} = \frac{(1-q^2)^2(1-s^2)^2}{(1-q^2)^2(1-s^2)^2 - 16s^2q^2},$$

where $s$ is a phase factor $s = \exp\left(i\frac{2\pi m_1{}'t}{\lambda' p}\right)$ and $q$ is as defined above for $m' = m_1{}'$. At peak reflectance ($\lambda' = 1$), the quadratic equation for $m'^2$ simplifies to

$$m'^2 = 2\left(1 \pm \sqrt{1+\left(\frac{\alpha}{2}\right)^2}\right)$$

In experimental systems, the birefringence parameter $\alpha$ is often small (e.g. for the related system of cellulose nanocrystals, $\alpha \approx 0.04$). The expression for $m'^2$ can therefore be expanded to lowest order in $\alpha$ as

$$m_1'^2 = 2\left(1-\left(1+\frac{1}{2}\left(\frac{\alpha}{2}\right)^2\right)+\cdots\right) \approx -\left(\frac{\alpha}{2}\right)^2.$$

Similarly, it can be shown that in this case $f_1 \approx \sqrt{-1}$ and $q_1^2 \approx -1$ so the peak reflectance is given by

$$R_{LCP} \approx \frac{(1-s^2)^2}{(1-s^2)^2 + 4s^2}.$$

As $m_1{}'$ is imaginary, the phase factor $s$ has the form $s = \exp(-z)$ for real $z$, and the LCP reflectance can be written more elegantly as

$$R_{LCP} = \tanh^2 z = \tanh^2\left(\frac{\pi\,\alpha\,t}{p}\right)$$

Finally, the birefringence $\alpha$ can be written explicitly in terms of refractive indices $n_j = \sqrt{\varepsilon_j}$ as

$$\alpha = \frac{n_2^2 - n_1^2}{n_2^2 + n_1^2}$$

and since $\alpha \ll 1$, this expression simplifies to $\alpha \approx \frac{\Delta n}{n_{avg}}$, where $\Delta n = n_2 - n_1$ and $n_{avg} = \sqrt{\varepsilon}$. Hence the LCP reflectance can be written as

$$R_{LCP} = \tanh^2\left(\frac{\pi\,\Delta n\,t}{n_{avg}\,p}\right).$$

For small reflectance values ($R_{LCP} \ll 1$), this equation simplifies to

$$R_{LCP} = \left(\frac{\pi\,\Delta n\,t}{n_{avg}\,p}\right)^2.$$

The equations for $R_{LCP}$ can also be inverted to give an expression for the birefringence of a domain of a given size and reflectance:

$$\Delta n = \frac{n\,p}{\pi\,t}\tanh^{-1}\sqrt{R_{LCP}} = \frac{n_{avg}\,p}{2\,\pi\,t}\ln\left(\frac{1+\sqrt{R_{LCP}}}{1-\sqrt{R_{LCP}}}\right).$$

It is important to note that the birefringence in the equations above is the effective birefringence of the helicoidal structure $\Delta n_{eff}$. For helicoidal ChNC films, the effective birefringence is related to the intrinsic

birefringence of the individual ChNCs $\Delta n_{ChNC}$ by the equation $\Delta n_{eff} = \Delta n_{ChNC}.S_2$, where $S_2$ is the nematic order parameter that quantifies the degree of local ordering and ranges from 0 to 1. For helicoidal structures made by colloidal self-assembly, $S_2$ is expected to be in the range 0.6-0.9,[2] so the intrinsic birefringence is expected to be no more than twice the effective birefringence.

## S3. Normalization of reflectance

Experimentally, the maximum measurable reflectance of a chiral nematic structure is dependent on the microscope setup and normalization convention. Reflectance values are typically normalized to a standard reference material; for instance, a silver mirror was used as reference for all spectra in this work. The LCP reflection from a sample was determined by illuminating the sample with unpolarized light and analyzing (i.e. filtering) the reflected light to select the LCP channel. The LCP reflectance, $R_{LCP}$, was therefore normalized to the LCP reflection from a silver mirror illuminated with unpolarized light. Since a perfect left-handed chiral nematic structure only reflects LCP light and no RCP light, the theoretical total reflectance, $R$, defined relative to unpolarized illumination, is equal to half the $R_{LCP}$, thus $R = \frac{1}{2} R_{LCP}$.

The reflection through crossed polarizers (XP) was measured by illuminating the sample with linear polarized light and analyzing the reflected light with a linear polarizer oriented perpendicular to the first polarizer. The XP reflectance, $R_{XP}$, was normalized to the reflection from a silver mirror between parallel polarizers. When illuminated with linearly polarized light, a perfect left-handed chiral nematic structure (with an associated $R_{LCP} = 1$) will reflect half of the incident light, as linearly polarized light is an equal mixture of LCP and RCP components and the chiral nematic will only reflect the LCP component. The linearly polarized analyzer (whether parallel or crossed relative to the first linear polarizer) then transmits only half of the reflected LCP light and thus only 25% of the incident (linearly polarized) light can reach the detector. The XP reflectance, normalized as described above, should therefore compare to $R_{LCP}$ as $R_{LCP} = 4\ R_{XP}$ (while under unpolarized light, we would get $R = 2\ R_{XP}$). In practice we found that the conversion factor between $R_{LCP}$ and $R_{XP}$ varied between 1 and 4, an observation that we ascribe to possible polarization effects of the beamsplitters within the microscope. To capture the uncertainty associated with this conversion factor, we provided our estimation of $\Delta n$ for a range of values between 1 and 4.

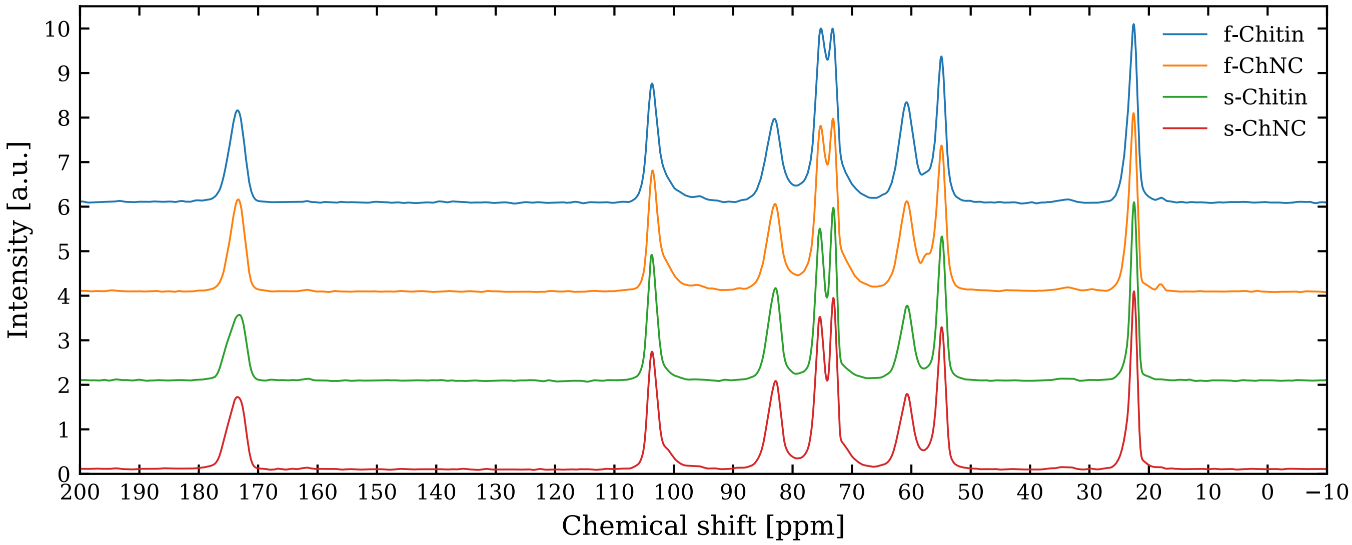


**Figure S1**. Stacked $^{13}C$-ssNMR spectra comparing fungal and shrimp chitin and the corresponding chitin nanocrystals (ChNCs). All four samples – fungal chitin (f-Chitin), fungal ChNC (f-ChNC), shrimp chitin (s-Chitin) and shrimp ChNC (s-ChNC) – correspond to the chitin structure and exhibit good purity.

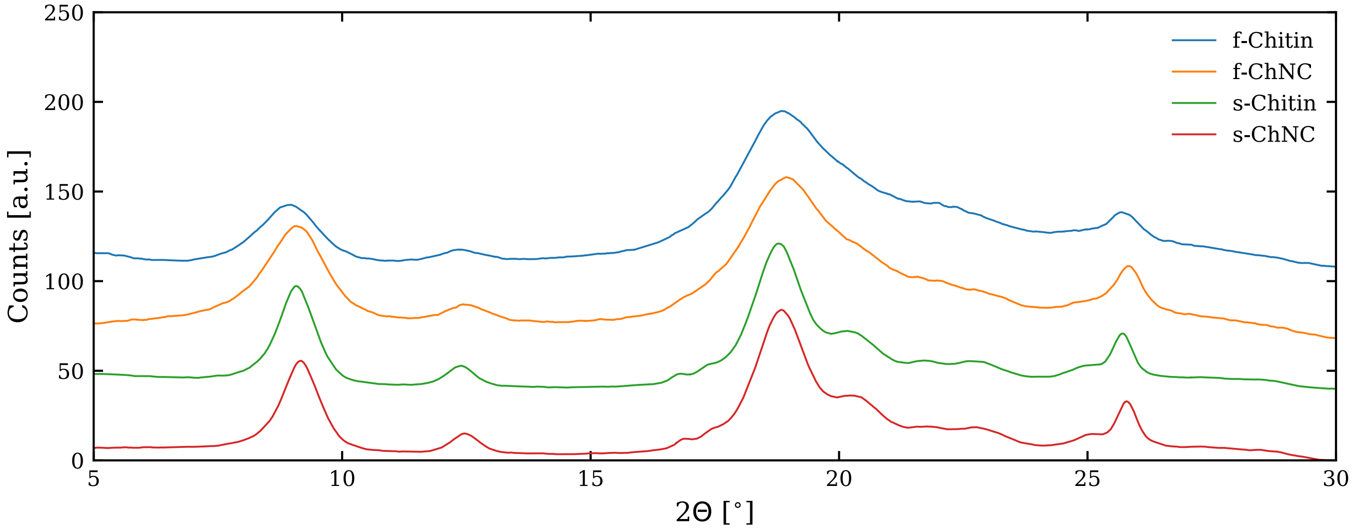


**Figure S2**. Stacked pXRD diffractograms comparing fungal and shrimp chitin and their corresponding ChNCs. The diffractograms indicate that there is little difference between chitin and the respective ChNCs produced, evidenced by the similar data for both pairs, i.e., fungal chitin (f-Chitin) and fungal ChNC (f-ChNC), and shrimp chitin (s-Chitin) and shrimp ChNC (s-ChNC). However, the fungi-derived materials show broader diffraction peaks suggesting that they are less crystalline than those derived from shrimp.

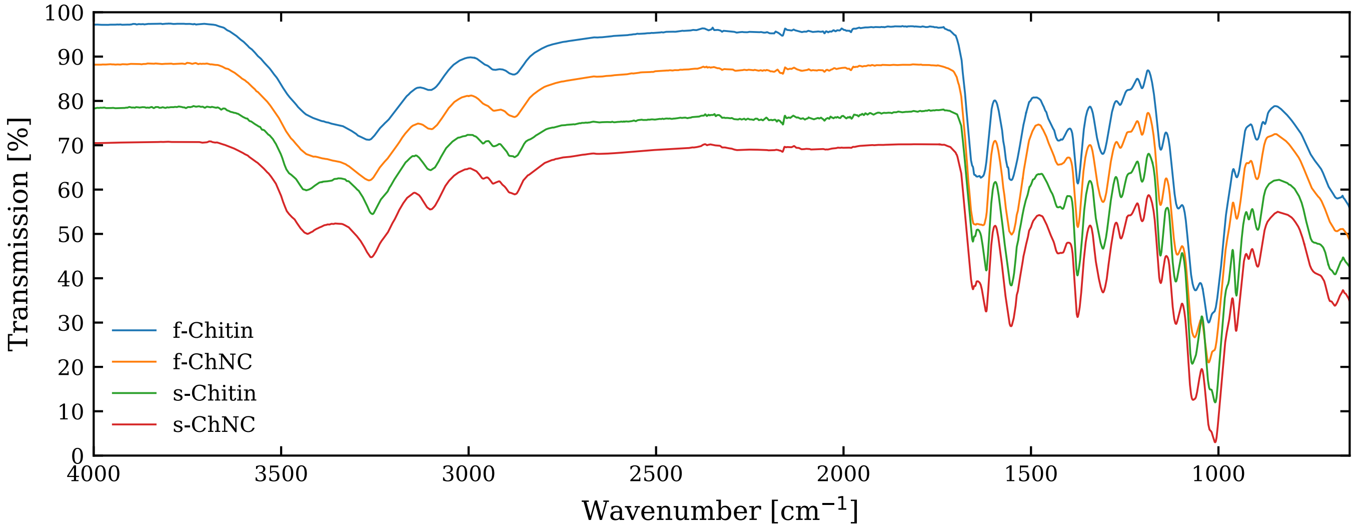


**Figure S3**. Stacked ATR-FTIR spectra comparing fungal and shrimp chitin and corresponding ChNCs. The two peaks at 1660 and 1620 $cm^{-1}$ correspond to the splitting of the amide carbonyl stretching signal which is associated with the α-chitin crystal structure. While it is very prominent for shrimp chitin (s-Chitin) and shrimp ChNC (s-ChNC) samples, the splitting is hard to resolve for fungal chitin (f-Chitin) and fungal ChNC (f-ChNC) samples. This suggests that fungal derived material is less crystalline than that originating from shrimp.

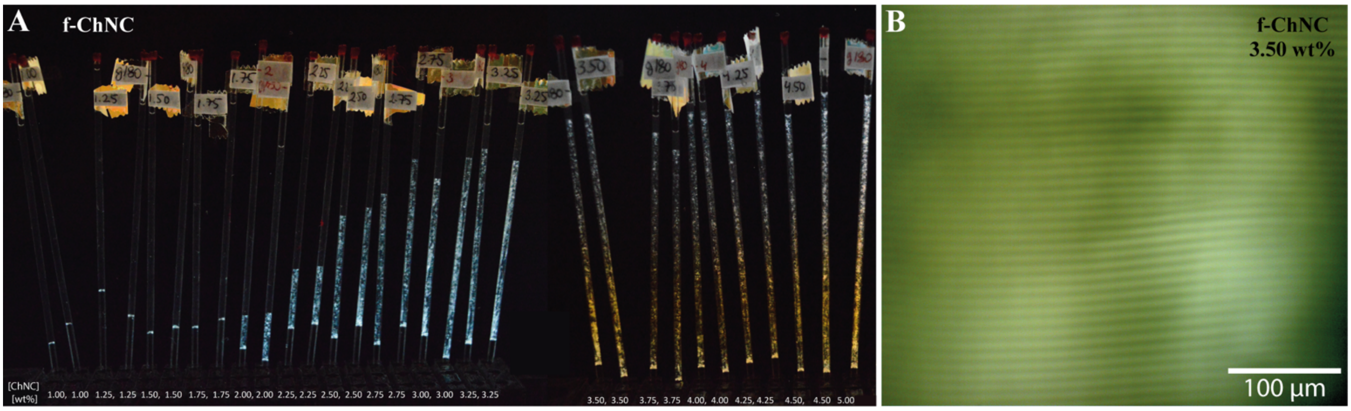


**Figure S4**. Self-assembly behavior of ChNCs derived from fungi. (**A**) Photograph of capillaries filled with f-ChNC suspension with increasing concentration. **(B)** The fingerprint pattern, characteristic for chiral nematic phase, was imaged by polarized optical microscopy for a 3.50 wt% ChNC suspension.

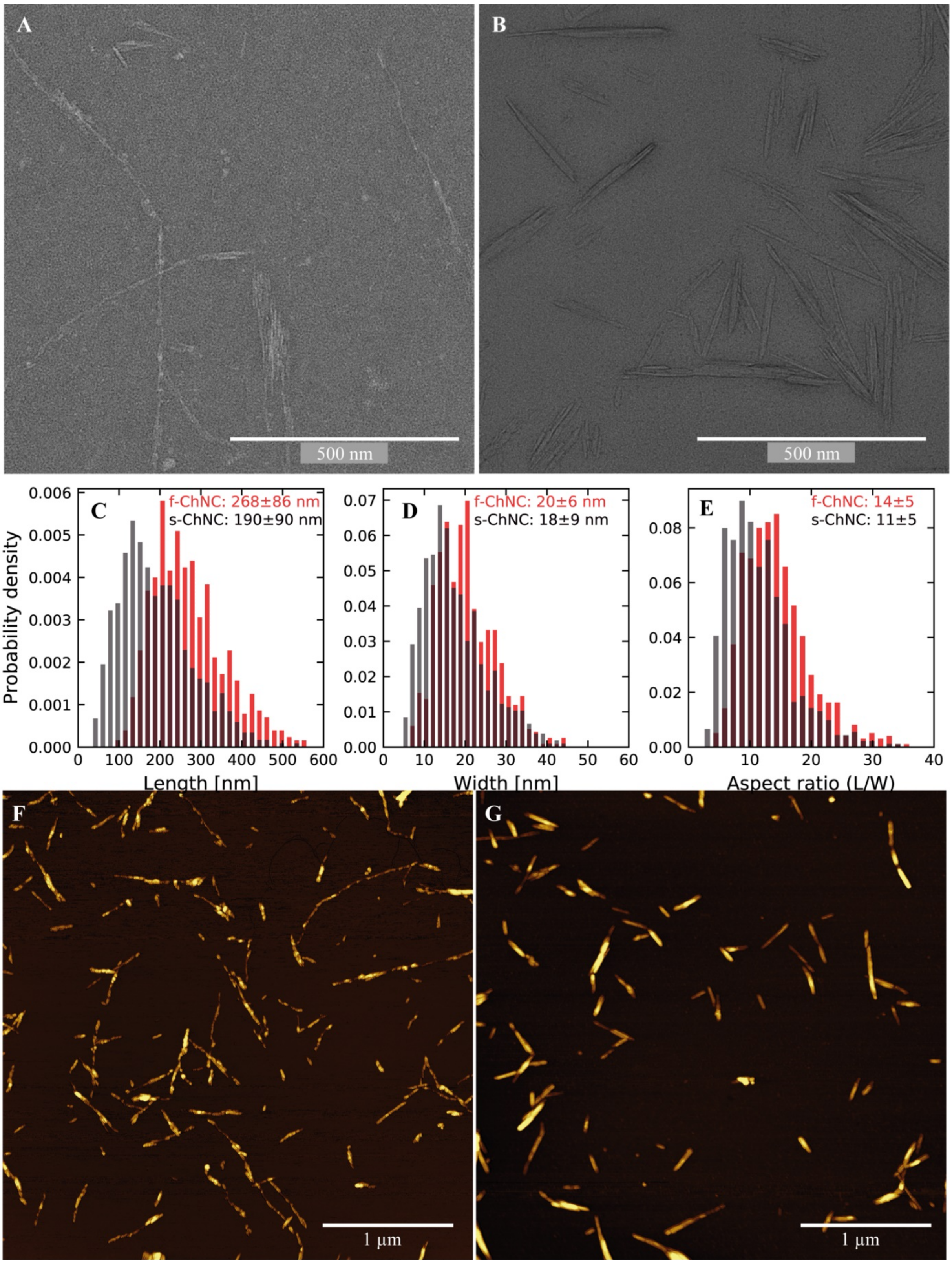


**Figure S5.** Representative transmission electron micrographs comparing the two sources: (**A**) f-ChNC and (**B**) s-ChNCs. The particles were analyzed using ImageJ software, by manually measuring particle dimensions to obtain statistics on (**C**) length and (**D**) width, allowing for (**E**) individual particle aspect ratios to be calculated. The average and standard deviation for each respective measurement is indicated in the top right corner of the histogram. These measurements reveal that f-ChNC are significantly longer with a larger aspect ratio than s-ChNCs, which agrees with the qualitative observations by atomic force microscopy (AFM), where (**F**) f-ChNC also appear much longer than (**G**) s-ChNC.

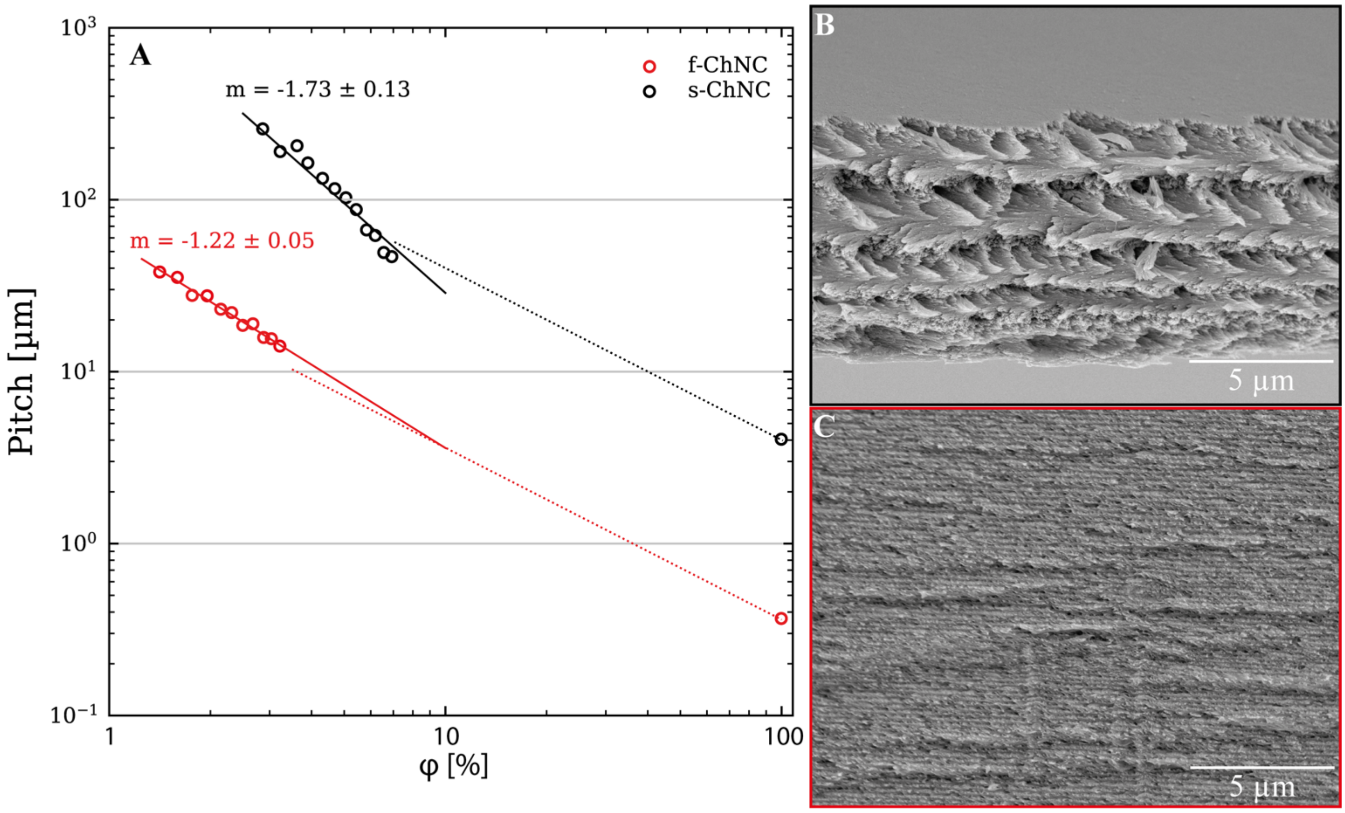


**Figure S6.** Pitch analysis of ChNCs self-assembled in suspension and after drying to form a film. (**A**) The chiral nematic pitch values at all concentrations except 100 vol% were obtained from the capillaries, (data presented in Figure 1, S4), and were replotted on a log-log scale with the ChNC concentration rescaled to be expressed in volume fraction. Data for s-ChNCs and f-ChNCs are denoted by black circles and red circles respectively. These suspensions are prepared by dialysis at 1 wt% against 0.6 mM HCl (see Experimental Methods). The evolution of the pitch is represented by the guide lines, with the point of kinetic arrest expected at their intersection. Note that pitch values at 100 vol% are measured by SEM of film cross-sections, with example micrographs provided in (**B**) for s-ChNC and (**C**) for f-ChNC.

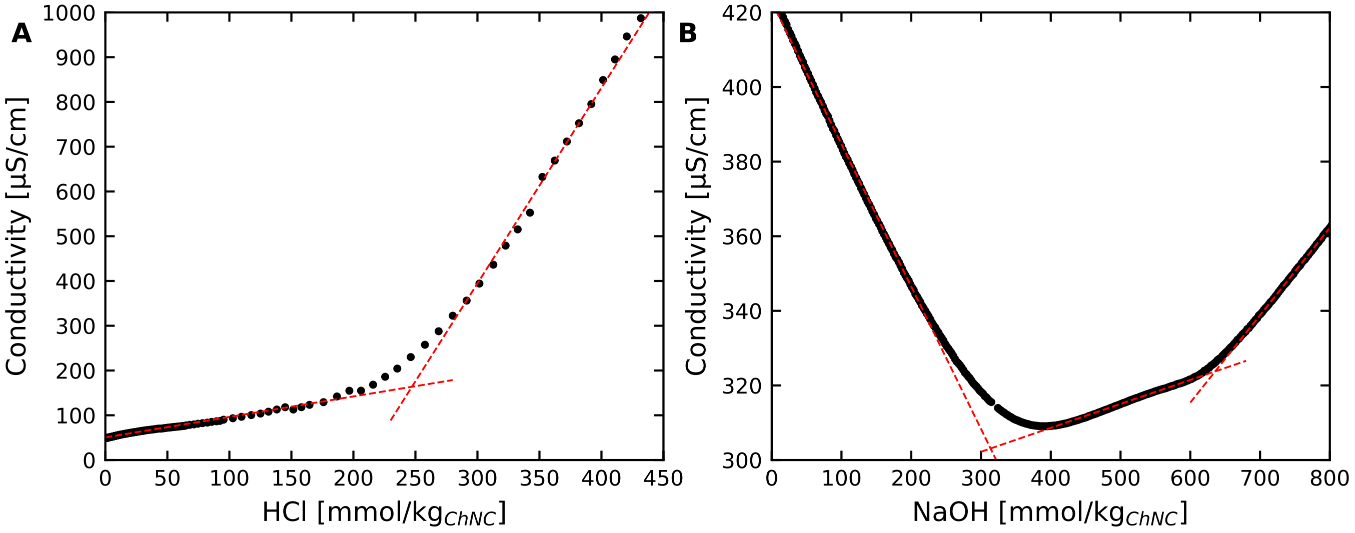


**Figure S7**. Titration of f-ChNC with HCl and NaOH. (**A)** Titration of f-ChNC suspension using HCl after extensive dialysis against Milli-Q water. Such a suspension can capture up to 246 mmol$_{HCl}$/kg before it becomes fully protonated, as determined from where the two lines intersect. This allows for the surface charge to be tuned without altering meaningfully the ionic strength **(B)** Titration of the same f-ChNC suspension using NaOH, starting with an initial excess of added HCl. This titration reveals that the maximum surface charge attainable for f-ChNC suspensions, given by the gap between the two equivalence points, is 330 mmol/kg, as determined from the intersection of the three lines fitted to the experimental data.

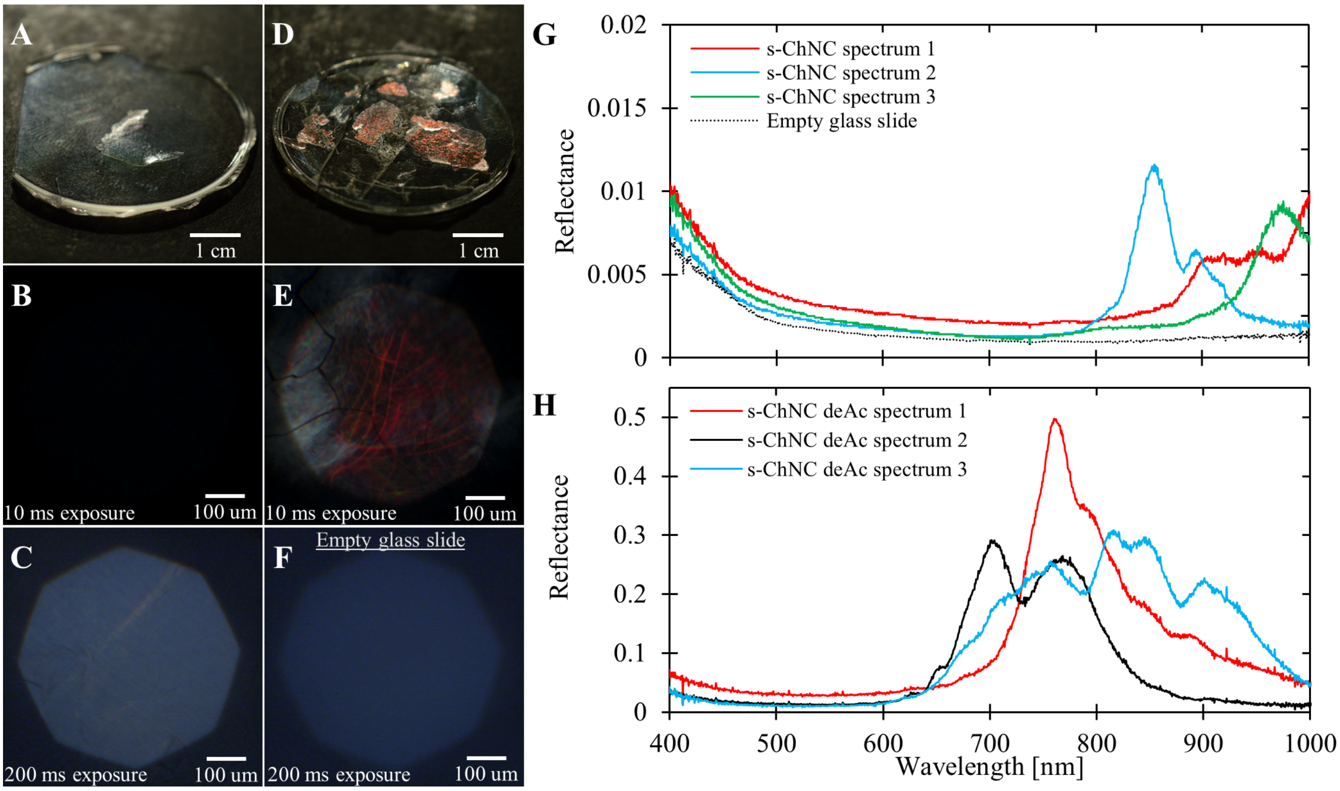


**Figure S8.** Pitch contraction after alkaline post-treatment. **(A)** Photograph of a flake from an apparently colorless s-ChNC film on a polystyrene Petri dish. The film was prepared by extensively dialyzing s-ChNC suspension against Milli-Q water, followed by adding 100 mmol/kg HCl and 120 mmol/kg NaCl. **(B, C)** Cross polarized optical micrographs of the film in (A) at different camera exposure times. (**D**) After an in-situ treatment with concentrated sodium hydroxide, the film becomes red, which is confirmed by optical microscopy in **(E)**. **(F)** POM micrograph obtained using identical conditions as in (C) but of a glass slide without a sample, showing that the blueish tint in (C) was a measurement artefact, also seen in (G) as the weak shoulder in the spectroscopy data (dotted line). (**G**) Three exemplary spectra from s-ChNC films being beyond the visible range. (**H**) Presentation of three typical spectra from s-ChNC flake from the same film after it has been treated with an alkali. The reflectance has increased around 20-fold and the signal has been shifted to lower wavelengths, explaining the presence of some red coloration. Nevertheless, most of the signal remains still outside visible spectrum.

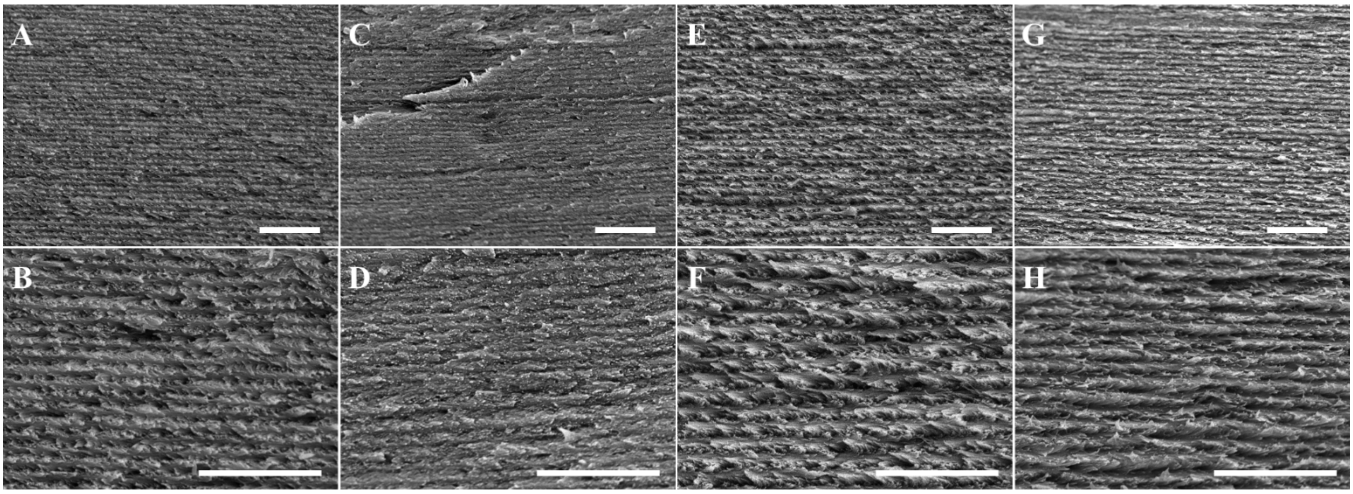


**Figure S9**. Cross-sectional SEM images of ChNC films before and after in-situ alkaline treatment. (**A, B**) f-ChNC film before alkaline treatment. (**C, D**) f-ChNC film after alkaline treatment (same film, different flake). (**E, F**) s-ChNC film before alkaline treatment. (**G, H**) s-ChNC film after alkaline treatment (same film, different flake). In both cases, the structure is retained with a noticeable reduction in pitch and overall film thickness. All scale bars are 2 μm.

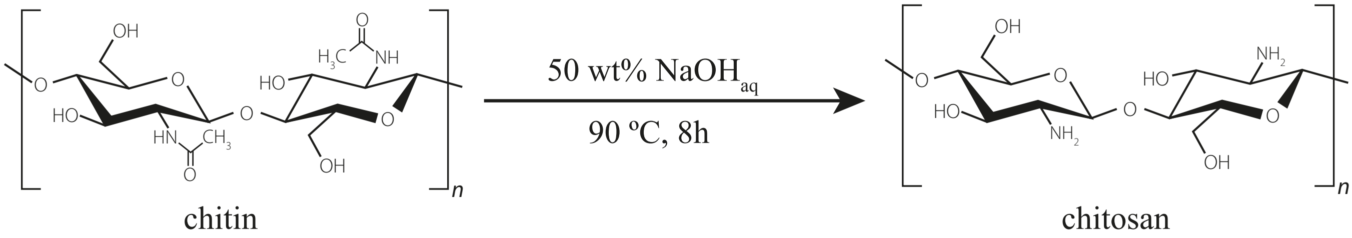


**Figure S10**. Schematic showing the hydrolysis of chitin to chitosan with concentrated sodium hydroxide.

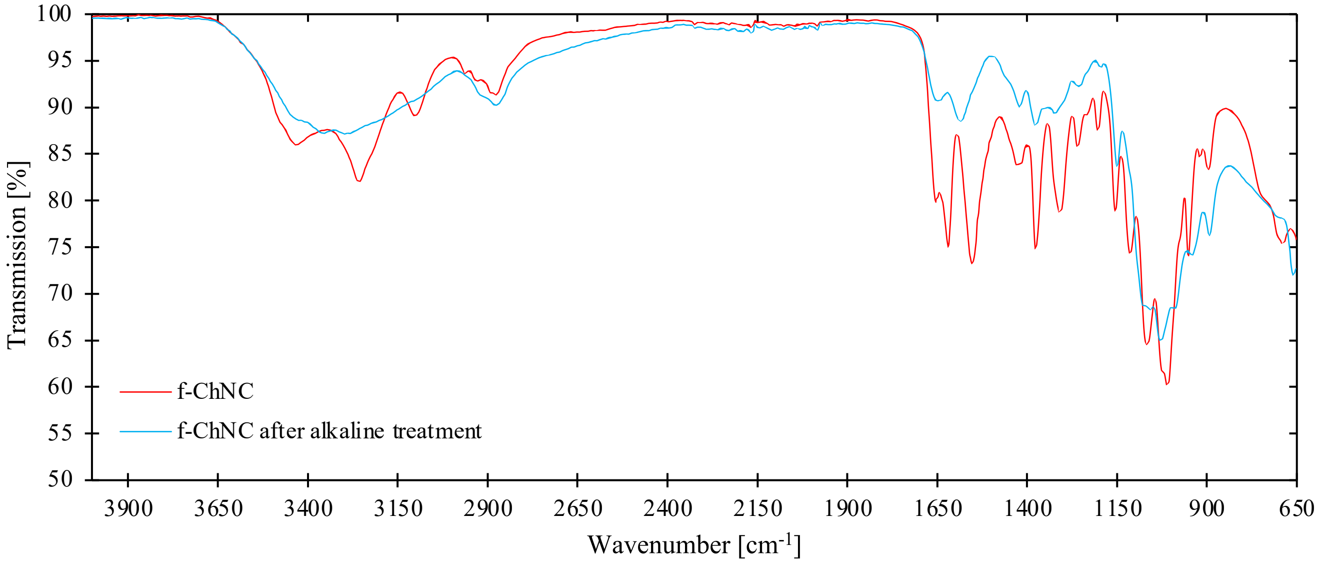


**Figure S11.** ATR-FTIR spectra of the fungal ChNC film before (*red*) and after (*blue*) alkaline treatment. The peaks at 1554, 1619, and 1656 $cm^{-1}$ are associated with the amide in α-chitin, which after the alkaline treatment have disappeared and are replaced by new peaks at 1585 and 1650 $cm^{-1}$. This indicates a successful conversion from chitin to chitosan.

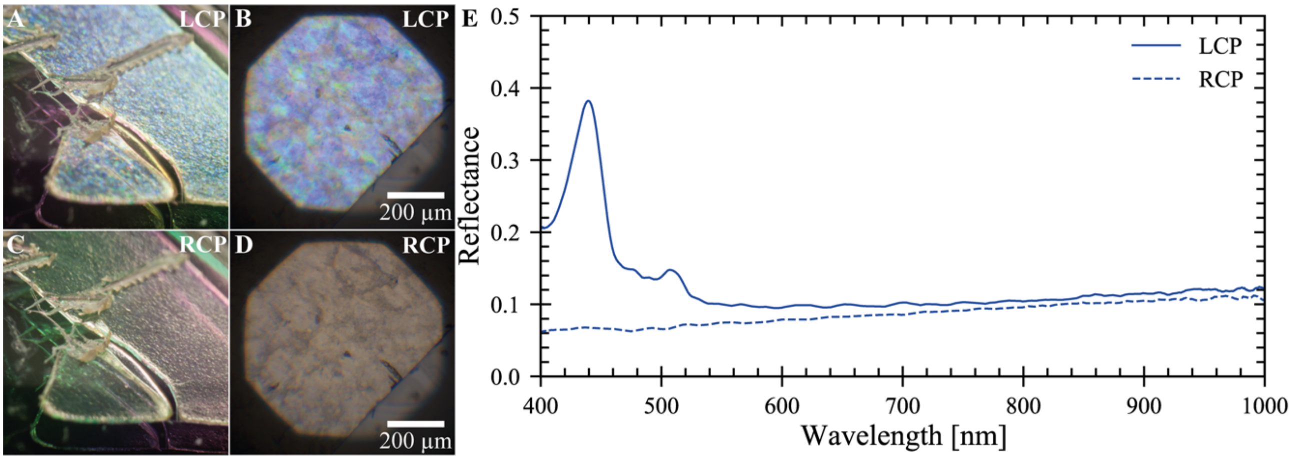


**Figure S12**. Increased reflectance after the alkaline treatment of solid-state f-ChNC films. (**A**) Macroscopically, structural coloration can be observed by naked eye when selecting for the reflected left circularly polarized (LCP) light and (**B**) when observing with optical microscopy. (**C, D**) The color disappears when collecting only right circularly polarized (RCP) light, consistent with the expected optical behavior of a left-handed helicoidal structure. (**E**) micro-spectroscopy confirms the selective reflection of LCP light.

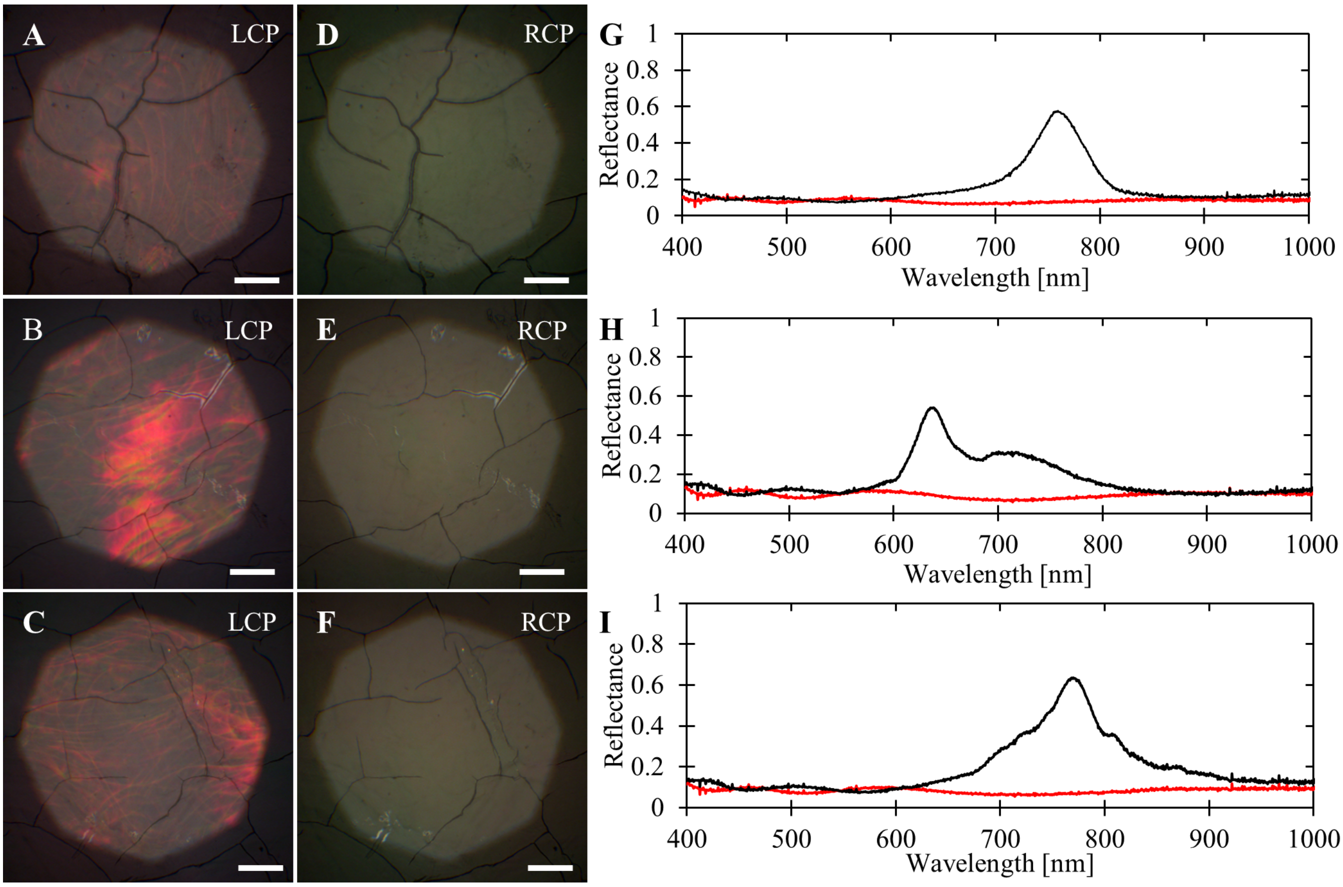


**Figure S13**. Polarized optical microscopy of s-ChNC films after alkaline treatment. Representative micrographs of s-ChNC films after alkaline treatment when viewed in reflection (**A, B, C**) collecting only left circularly polarized light (LCP), and (**D, E, F**) only right circularly polarized light (RCP). Scale bars are 1 µm. (**G, H, I**) Corresponding micro-spectroscopy for LCP light (black line, clear peak) and for RCP (red line, no peak). The baseline offset (*ca.* 0.1) originates from the reflection at the air/chitin interface.

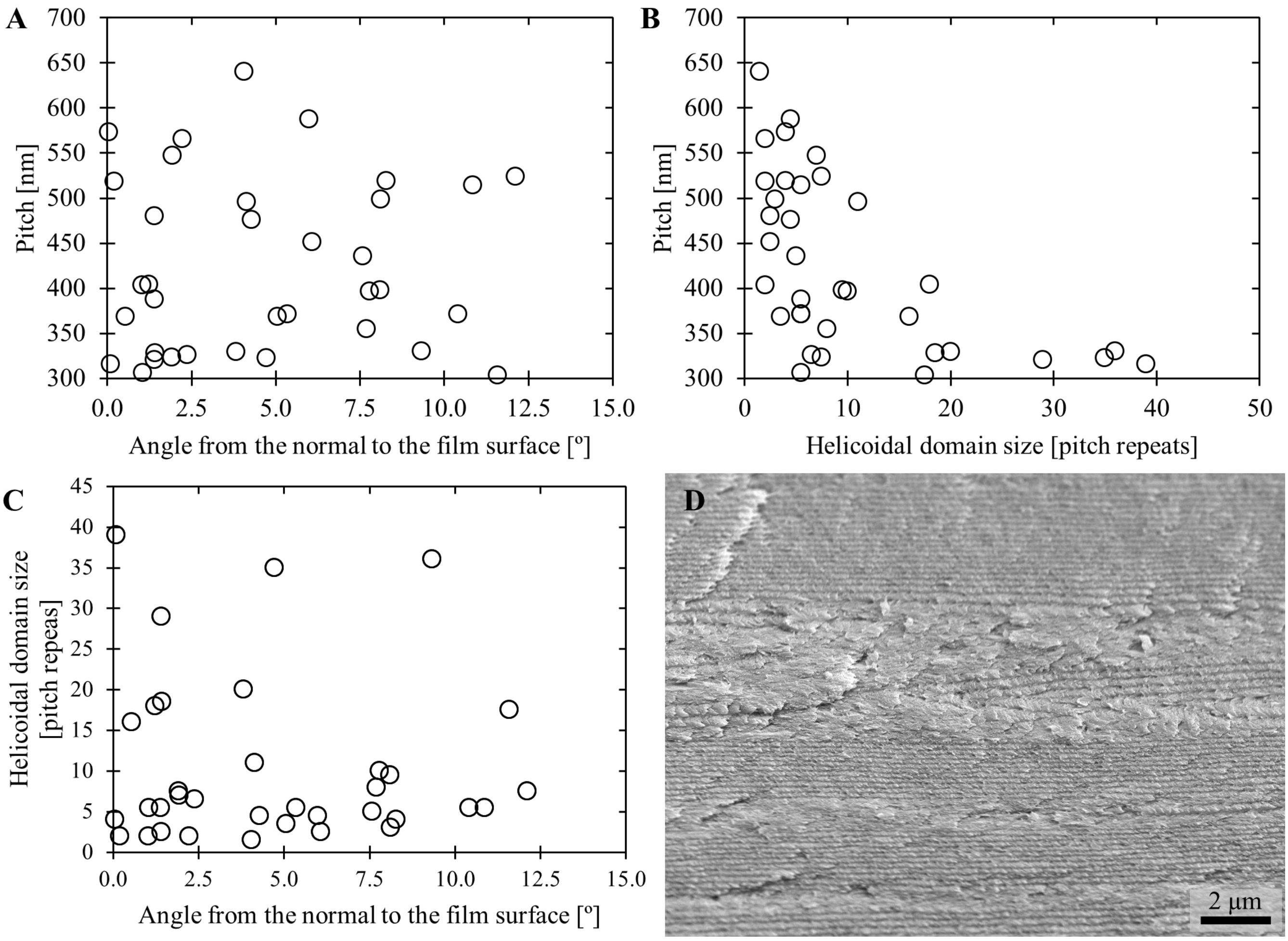


**Figure S14.** The pitch distributions for helicoidal domain: (**A**) Plotted against the helicoidal domain angle, as defined with respect to the line perpendicular to the film surface. (**B**) Plotted against the helicoidal domain size, as expressed in pitch repeats. (**C**) Helicoidal domain size plotted against the helicoidal domain angle. Values were measured from the cross-sectional SEM images for an f-ChNC film. (**D**) Example of a misaligned helicoidal domain seen by cross-sectional SEM image of an f-ChNC film.

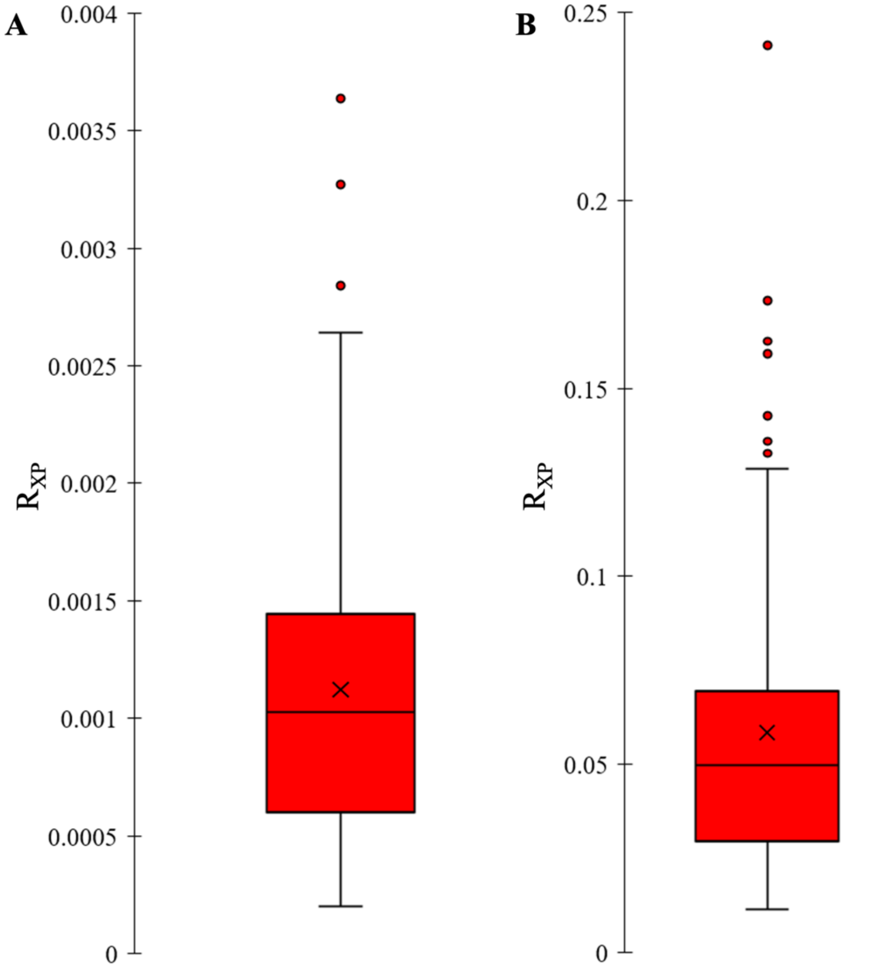


**Figure S15**. A statistical distribution of the maximal reflection of the peaks of a f-ChNC film (A) before and (B) after alkaline post-treatment. The "×" marks the mean value. The horizontal line marks the median line. The error bars mark the standard deviation. Circles mark the outliers. When determining maximal reflection, the background reflection was removed. The data was obtained from at least 90 individual spectra. $R_{XP}$ denotes reflection measured between crossed polarizers.

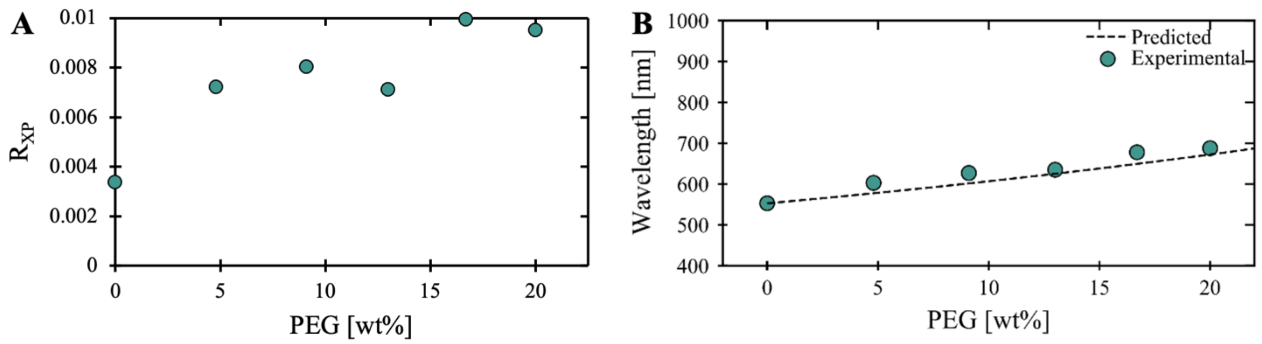


**Figure S16**. Effect of the addition of PEG within ChNC films, in terms of (**A**) peak reflectance and (**B**) peak wavelength from cross polarized (XP) optical micro-spectroscopy. The latter is compared to predictions for a non-volatile and chirally non-interacting additive (dotted line). Poly(ethylene glycol) red-shifts the reflected color, which is consistent with previous observations on structurally colored cellulose nanocrystal films.[3]

[1] H. D. Vries, *Acta Crystallogr.* **1951**, *4*, 219.
[2] C. Honorato-Rios, A. Kuhnhold, J. R. Bruckner, R. Dannert, T. Schilling, J. P. F. Lagerwall, *Front. Mater.* **2016**, *3*, 21.
[3] K. Yao, Q. Meng, V. Bulone, Q. Zhou, *Adv. Mater.* **2017**, *29*, 1701323.
